# Genetic heterogeneity of swine hepatitis E virus isolates from Yunnan province, China in 2011–2012

**DOI:** 10.1186/1743-422X-11-162

**Published:** 2014-09-04

**Authors:** Xianghua Shu, Xinhui Duan, Chunlian Song, Jintao Li, Lei Jiang, Gefen Yin, Wengui Li

**Affiliations:** College of Animal Science and Technology, Yunnan Agricultural University, Kunming, 650201 China; Department of Laboratory Animal Science, Kunming Medical University, Kunming, 650500 China

**Keywords:** Hepatitis E virus, Phylogenetic analysis, Viral diversity

## Abstract

**Background:**

Hepatitis E is a disease of major public-health concern mainly in developing countries. Although molecular and sero-epidemiological investigations of HEV have been performed in many provinces in China, the epidemiological data from Yunnan Province are limited and genotypes are not be fully characterized. In this study the prevalence and characteristics of hepatitis E virus (HEV) detected in pigs from Yunnan province, China was evaluated.

**Results:**

A total of 13 out of 187 pig fecal samples collected in 2011 revealed HEV positive results; likewise, 7 out of 69 samples collected in 2012 exhibited positive results. These findings indicated a total prevalence of 7.8% (20/256). Phylogenetic and molecular evolutionary analysis results revealed that nine strains were found in the samples obtained in 2011, in which 87.1% to 99.4% nucleotide sequence identity was shared among these strains; and 77.0% to 81.9%, 52.2% to 53.6%, 77.0% to 88.2% and 77.9% to 96.8% nucleotide sequence identities were shared with strains representing genotypes 1, 2, 3, and 4. Five strains were detected in the samples obtained in 2012, in which 94.2% to 99.3% nucleotide sequence identity was shared among the strains, and 81.0% to 82.5%, 81.8% to 83.2%, 81.0% to 92.7% and 81.0% to 97.8% nucleotide sequence identities were shared with strains representing the genotypes 1, 2, 3, and 4.

**Conclusions:**

Analysis of fourteen detected HEV strains revealed that three of them were subtype 4d, two were subtype 4b; the nine remaining isolated strains were subtype 4 h. These results indicated that the prevalence of HEV in the swine herds of Yunnan was quite high, additional public-health concerns should focus on pork safety.

**Electronic supplementary material:**

The online version of this article (doi:10.1186/1743-422X-11-162) contains supplementary material, which is available to authorized users.

## Background

Hepatitis E, caused by hepatitis E virus (HEV), is the most frequent cause of acute hepatitis, acute liver failure, and acute-to-chronic liver failure in humans. Hepatitis E causes high morbidity and mortality in patients with underlying liver disease; this disease can also progress into chronic infection that causes fibrosis in immunocompromised hosts [1]. Based on seroprevalence data the World Health Organization (WHO) estimated that at least one-third of the world population, residing mainly in Asia, Africa, Middle East, and Central America exhibits history of HEV infection [2].

HEV is the sole member of the genus Hepevirus in the family Hepeviridae. The genome is a single-stranded, positive-sense RNA molecule of approximately 7.2 kb in size. HEV is genetically diverse, although all of the mammalian HEV isolates possibly belong to a single serotype. The known HEV sequences have been analyzed, revealing at least four major genotypes (genotypes 1 to 4). G1 and G2 are transmitted from one human to another, and these genotypes are often associated with outbreaks or large epidemics in developing countries; G3 and G4 are zoonotic, in which swine and other animals function as reservoir of human HEV infections. With the identification of infectious HEV in meat and meat products and resultant sporadic cases of food-borne hepatitis E in human populations, food safety associated with HEV contamination has been considered as an important public health concern [3, 4], particularly in developing countries where sporadic cases have been increasingly documented [5].

Although molecular and sero-epidemiological investigations of HEV have been performed in many provinces in China, the epidemiological data from Yunnan Province are limited; genotypes are yet to be fully characterized. The study aimed to investigate the prevalence of HEV infection among pigs and determine the extent of genetic variations in Yunnan HEV strains by phylogenetic and molecular evolutionary analyses.

## Results

### RT-nPCR detection

Among the 187 fecal samples collected in 2011, 13 (7.0%) were positive for HEV RNA after RT-nPCR detection was performed; among the 69 samples collected in 2012, 7 (10.1%) positive samples were found. All of the 20 products were sequenced and aligned. Five of the thirteen strains detected in 2011 and three of the seven strains detected in 2012 shared identical sequences but were removed. Fourteen sequences were identified in this study. Nine sequences detected in 2011 were deposited in GenBank: YN11-1 (JN542514), YN11-2 (JN542515), YN11-3 (JN542516), swYN11-4 (JN613152), swYN11-5 (JN613153), swYN11-6 (JN613154), swYN11-7 (JN613155), swYN11-8 (JN613156), and swYN11-9 (KF703731). Five sequences (swKM12-2, swKM12-3, swKM12-4, swKM12-5, and swKM12-6) detected in 2012 were unacceptable in GenBank because these sequences were < 200 bp in length (Additional file 1).

### Phylogenetic analysis of PCR products

The phylogenetic and molecular evolutionary analyses revealed that nine strains detected in 2011 shared 87.1% to 99.4% nucleotide sequence identity with one another; these results also revealed 77.0% to 81.9%, 52.2% to 53.6%, 77.0% to 88.2%, and 77.9% to 96.8% nucleotide sequence identities with selected strains representing G1, G2, G3, and G4. Five strains detected in 2012 shared 94.2% to 99.3% nucleotide sequence identity with one another, and 81.0% to 82.5%, 81.8% to 83.2%, 81.0% to 92.7%, and 81.0% to 97.8% nucleotide sequence identities with strains representing G1, G2, G3, and G4.

**Figure 1:**
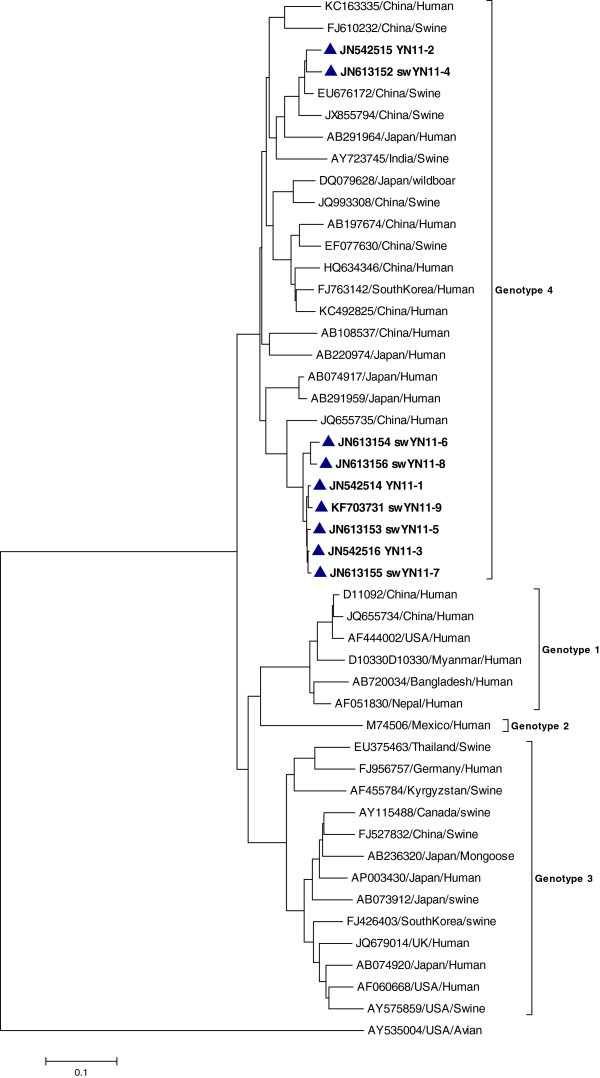
**Phylogenetic tree constructed by aligning the swine HEV strains detected in 2011.** Analyses based on nine isolated strains and 38 full length genomic references sequences. The tree was constructed by the Neighbor joining method using MEGA 5.05. Reference sequences are labeled with the GenBank accession number, the Country and host of the strain was isolated.

**Figure 2:**
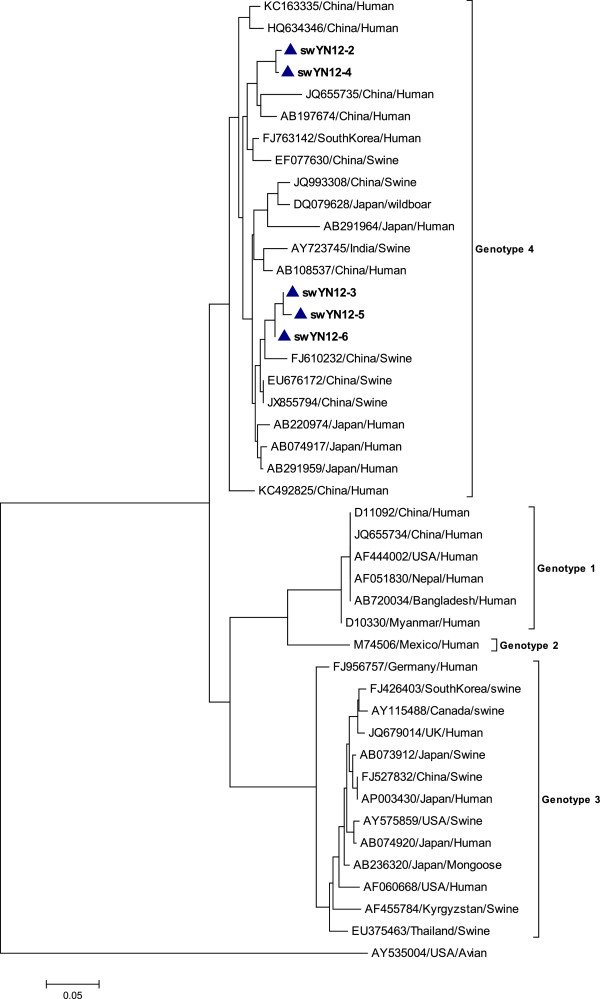
**Phylogenetic tree constructed by aligning the swine HEV strains detected in 2012.** Analyses based on five isolated strains and 38 full length genomic references sequences. The tree was constructed by the Neighbor joining method using MEGA 5.05. Reference sequences are labeled with the GenBank accession number, the Country and host of the strain was isolated.

Two phylogenetic trees, one for the strains detected in 2011 (Figure 1) and another for the strains detected in 2012 (Figure 2), were constructed using the neighbor-joining method based on isolate sequences and 38 reference HEV sequences from China and other countries. Figure 1 shows that one branch includes seven strains, which were isolated from Yunnan. The strains detected in 2011 clustered with HEV strains isolated in Wuhan City and other prevalent strains in China.

### Genotype and subgenotype analyses

Our results revealed that all of the 14 isolates belonged to G4 HEV and clustered with China swine and human HEV sequences. Subtype analysis results revealed that most of the sequences (71.4%, 9/14) were subtyped as 4 h, three (swYN12-3, swYN12-5, and swYN12-6) were subtyped as 4d and two (YN11-2, and swYN11-4) were subtyped as 4b. Phylogenetic analysis results showed that a distinct G4 lineage (4 h) is circulating in Yunnan Province. Amino acid analysis results revealed unique mutations of F3 → L3, R34 → C34, S46 → P46, D77 → G77, and G87 → S87 in nine strains detected in 2011 and P21 → S21, A24 → T24, and V27 → A27 in five strains detected in 2012.

## Discussion

Various HEV genotypes exhibit different modes of transmission, non-human reservoirs, and abilities of interspecies transmission, although HEV is mainly transmitted in water and food. G3 and G4 are known as causative agents of zoonotic diseases; thus researchers have been prompted to determine the reason that G3 and G4 can cross species barriers, but G1 and G2 strains cannot [6]. G3 HEV is mainly prevalent in humans of certain industrialized nations and has also been isolated from domestic and wild swine, deer, mongoose, rats, and rabbits. Whereas, G4 HEV is associated with sporadic cases of hepatitis E in humans and infects both wild and domestic swine; G4 HEV is also reportedly detected in cattle and sheep [3, 7]. Previous studies have suggested that the zoonotic transmission of HEV across several species, such as humans, pigs, boars, deer, chickens, and rabbits, may be the major mode of infection in non-endemic areas. Contact with swine is the most widely recognized route of occupational exposure to HEV, and humans have been considered as a major source of HEV in endemic areas [8, 9]. Epidemiological patterns also differ significantly between regions where this disease is highly endemic. A recent study in China even found that the seasonal changes in the prevalence of HEV in swine may be attributed to the geographical distribution of different subtypes [10].

Studies have been conducted regarding HEV transmission in non-human primates such as cynomolgus, rhesus, owl monkeys, and chimpanzees, as well as in pigs, rabbits, and Mongolian gerbils [11]. Experimental HEV infections in animal models have provided relevant information regarding the biological characteristics and pathogenesis of HEV; these animal models are also essential tools used in vaccine and drug test [12]. However, effective tissue culture replication systems of HEV have rarely been developed [13]. Furthermore, the pathogenesis of liver pathology and the replication cycle of HEV remain poorly understood because cultured cells are unable to propagate efficiently in vitro.

G4 is responsible for the majority of sporadic hepatitis E cases affecting humans in China, and the high prevalence of G4 in pig population exacerbates this situation. G4-induced disease symptoms are possibly more severe than other types. In a previous study involving putative HEV G4 virulence determinants, one potential determinant is located in each of the 5ʹ-UTR and 3ʹ-UTR, 3 and 12 determinants are detected in ORF1 and ORF2, respectively, and two determinants are found in the junction [14]. Thus far, at least nine subtypes (4a–4i) of G4 HEV isolates have been identified; numerous new subgenotypes have been reported in humans and pigs [15–18]. In one of our previous surveys, at least four subgenotypes (4c, 4d, 4b, and a new subgenotype) are prevalent in Yunnan Province. Five of the nine known subgenotypes of G4 have been identified as prevalent in Yunnan Province. Furthermore, subtype 4 h is dominant and has been isolated from a human patient with liver failure in south of China [19].

The lack of a standardized assay for clinical diagnosis remains a challenging issue, thus HEV has been considered as an underreported pathogen of acute hepatitis cases. The diagnosis of HEV infection should depend on RT-PCR and serology. The majority of HEV RT-PCR assays used for diagnosis have been developed by choosing different conserved HEV genomic regions as a target for amplification and primers and probes should also be designed to guarantee the development of highly sensitive and broadly reactive assays because of the wide genetic heterogeneity of the HEV genome [20]. In general, G4 exhibits the lowest nucleotide sequence identity with G2 but higher nucleotide sequence identity in the same genotype. At present, HEV infections are serologically diagnosed by ELISA. The recombinant human HEV capsid antigen undergoes cross-reaction with antibodies to swine HEV in ELISA and has been widely used to detect anti-HEV antibody in swine [10].

A majority of infections in animals are asymptomatic and have not caused any economic loss in pig farms. As a result, the high prevalence of HEV in swine population cannot attract active management from farmers or authorities. Previous study results indicated that sustained transmission could induce changes in virulence; as such, severe consequences may occur [4]. Meanwhile, the number of published HEV sequences has increased significantly, analysis of genomic sequences of multiple HEV isolates have revealed extensive genomic diversity. Since the development of the first vaccine to prevent hepatitis E infection of humans registered in China in 2011, an effective method to control hepatitis E has been established. Nevertheless, HEV infection should also be controlled in reservoir animals, particularly in swine. Further studies should also be conducted to determine the duration of protection from vaccination, zoonotic transfer mode, difference in virulence between genotype and subgenotype, and vaccine for host animals.

## Conclusions

G4 HEV is highly prevalence in Yunnan Province of China and 4 h is likely the predominant subtype. Authorities should raise public-health concerns related to pork safety and risk of HEV infection via the consumption of undercooked pork products.

## Materials and methods

### Sample collection

Fresh swine fecal samples were collected from the piglets in markets and 3-6 month pigs on farms around Kunming, the capital city of Yunnan Province. All of the samples were stored at –80 °C until use. In 2011, a total of 187 swine fecal samples were collected from three counties; in 2012, 69 samples were collected from two counties (Table 1).

**Table 1:** **Sampling sites and HEV detection results**

Sampling sites	No. of samples	Isolation time	Positive rate	Isolated strains
Luquan County (piglet market)	66	July, 2011	4.5%(3/66)	YN11-1
JiangChuan County (back yard)	100	May, 2011	8%(8/100)	YN11-2,YN11-3,swYN11-4,swYN11-5,swYN11-6,swYN11-7
Fumin County (back yard)	21	March, 2011	9.5%(2/21)	swYN11-8, swYN11-9
Fumin County (piglet market)	29	October 2012	6.8%(2/29)	swYN12-2
Shilin County (pig Farms)	40	October 2012	12.5%(5/40)	swYN12-3,swYN12-4,swYN12-5,swYN12-6
Total	256		7.8%(20/256)	

Five counties around Kunming, the capital city of Yunnan Province were selected for sampling. Fecal samples were collected from the piglets in markets and 3-6 month pigs on farms. Samples size, collected time and HEV detection results were also recorded.

### RNA extraction and reverse transcription-nested PCR

To detect HEV infection by reverse transcriptase nest polymerase chain reaction (RT-nPCR), we used nested universal primers, forming 348 bp products [8]. In 2012, the prevalence of HEV strains in 2011 was significantly divergent; as such, the primers used were replaced with more sensitive primers that can amplify all of the known HEV sequences at that time [9]. Total RNA was extracted from 100 μl of stool suspension according to the manufacturer’s instructions. RT-nPCR was then performed according to previously described methods [12].

### Sequencing and phylogenetic analysis

The second-round PCR products were purified using a PCR product purification kit and ligated into pMD18-T vectors (Takara, Dalian, China). The plasmid was then used to transform *Escherichia coli* DH5α. Afterward, plasmids were extracted and the inserts were sequenced at Sangon Biological Engineering Company (Shanghai, China). Consensus sequences were aligned using DNAman (version 6.0, Lynnon Corporation). The nucleotide sequence identity between isolated sequences and four genotypes were calculated by using the Lasergene sequence analysis tool MegAlign (DNASTAR, Inc.). The phylogenetic and molecular evolutionary genetics analyses were conducted using the neighbor-joining method with MEGA 5.05 [21]. A total of 38 related HEV strains (Table 2) were used as references in the analyses; an avian HEV strain (AY535004) was included as an outgroup.

**Table 2:** **Reference HEV sequences used in the phylogenetic analyses**

Genotype	Strain	Host	Country	Accession No.
I	E11-Ban10	Human	Bangladesh	AB720034
	W2-1	Human	China	JQ655734
	C1	Human	China:Xinjiang	D11092
	HEVNE8L	Human	Myanmar	D10330
	TK15/92	Human	Nepal	AF051830
	pSK-HEV-2	Human	USA	AF444002
II	M	Human	Mexico	M74506
III	Arkell	swine	Canada	AY115488
	SAAS-JDY5	swine	China:Shanghai	FJ527832
	WB1-Aichi	wild boar	Japan: Aichi	DQ079628
	JMNG-Oki02C	mongoose	Japan: Okinawa	AB236320
	JMY-Haw	Human	Japan: Sapporo	AB074920
	SwJ570	swine	Japan:Tochigi	AB073912
	JRA1	Human	Japan:Tokyo	AP003430
	Osh-205	swine	Kyrgyzstan: Osh	AF455784
	Kernow-C1	Human	United Kingdom	JQ679014
	pSHEV-3	swine	USA	AY575859
	HEV-US1	Human	USA	AF060668
	HEV_RKI	Human	Germany	FJ956757
	Thai-swHEV07	Swine	Thailand	EU375463
IV	JYI-ChiSai01C	Human	China:Shanghai	AB197674
	SS19	swine	China:Guangdong	JX855794
	swGX40	swine	China:Guangxi	EU676172
	Ch-S-1	swine	China:Jilin	EF077630
	CH-YT-1	Human	China:Shangdong	KC163335
	CH-YT-HEV02	Human	China:Shangdong	KC492825
	TW6196E	Human	China:Taiwan	HQ634346
	IND-SW-00-01	Swine	India	AY723745
	HE-JA2	Human	Japan: Hokkaido	AB220974
	JTC-Kit-FH04L	Human	Japan:Hokkaido	AB291959
	JYK-Tok03C	Human	Japan:Tokyo	AB291964
	swKOR-1	swine	South Korea	FJ426403
	KNIH-hHEV4	Human	South Korea	FJ763142
	JKK-Sap	Human	Japan: Sapporo	AB074917
	W3	Human	China	JQ655735
	CCC220	Human	China:Jilin	AB108537
	HEV-ZJ1	Swine	China:Jiangsu	JQ993308
	swCH189	Swine	China: Gansu	FJ610232
		Avian	USA	AY535004

All references are full length genomic sequences, for each reference, the genotype, name, host, isolated country and accession number were recorded.

## Electronic supplementary material

Additional file 1: **Five sequences (swKM12-2, swKM12-3, swKM12-4, swKM12-5, and swKM12-6) detected in 2012.** These sequences were unacceptable in GenBank because of < 200 bp in length. (DOC 16 KB)
