# Supplementary material for: Genetic heterogeneity of swine hepatitis E virus isolates from Yunnan province, China in 2011–2012
Source: Virol J. 2014 Sep 4;11:162. doi: 10.1186/1743-422X-11-162 (PMC4163165; doi:10.1186/1743-422X-11-162)
Supplement: Supplementary file 1 — Additional file 1: Five sequences (swKM12-2, swKM12-3, swKM12-4, swKM12-5, and swKM12-6) detected in 2012. These sequences were unacceptable in GenBank because of < 200 bp in length. (DOC 16 KB) [file 12985_2014_2487_MOESM1_ESM.doc]

Five sequences (swKM12-2, swKM12-3, swKM12-4, swKM12-5, and swKM12-6) detected in 2012. These sequences were unacceptable in GenBank because of < 200 bp in length.

swYN12-2

1 GATTGGTCTC GCCAAGTGGA ACCGAGTGGG CGGGCTGGTT GTCGAGGGCG AGCTCCAGCC

61 CCGGCGGTGG CTGAAATGTC AGATGCGAAG GGGTTGGTTG GATGAATATA GGGGAGGGCG

121 AAGGGCTGAG AATCAAC

VDSQPFALPYIHPTNPFASDISATAGAGARPRQPARPLGSTWRDQ

swYN12-3

1 GATTGGTCTC GCCAAGTGGA ACCGAGTGGG CGGGCTGGCT GCCGAGGGCG AGCTCCAACC

61 CCGGCTGCGG CTGGAATGTC AGATGCGAAG GGGTTGGTTG GATGAATATA GGGGAGGGCG

121 AAGGGCTGAG AATCAAC

VDSQPFALPYIHPTNPFASDIPAAAGVGARPRQPARPLGSTWRDQ

swYN12-4

1 GATTGGTCAC GCCAAGTGGA ACCGAGTGGG CGGGCTGGTT GTCGAGGGCG AGCTCCAGCC

61 CCGGCGGTGG CTGAAATGTC AGATGCGAAG GGGTTGGTTG GATGAATATA GGGGAGGGCG

121AAGGGCTGAG AATCAAC

VDSQPFALPYIHPTNPFASDISATAGAGARPRQPARPLGSTWRDQ

swYN12-5

1 GATTGGTCTC GCCAAGTGGA ACCGAGTGGG CGGGCTGGCT GCCGGGGGCG AGCTCCAACC

61 CCGGCTGCGG CTGGAATGTC AGATGCGAAG GGGTTGGTTG GATGAATATA GGGGAGGGCG

121 AAGGGCTGAG AATCAAC

VDSQPFALPYIHPTNPFASDIPAAAGVGARPRQPARPLGSTWRDQ

swYN12-6

1 GATTGGTCTC GCCAAGTGGA ACCGAGTGGG CGGGCTGGCT GCCGAGGGCG AGCTCCAGCC

61 CCGGCTGCGG CTGGAATGTC AGATGCGAAG GGGTTGGTTG GATGAATATA GGGGAGGGCG

121 AAGGGCTGAG AATCAAC

VDSQPFALPYIHPTNPFASDIPAAAGAGARPRQPARPLGSTWRDQ
